# Increased IgA-mediated responses to the gut paracellular pathway and blood–brain barrier proteins predict delirium due to hip fracture in older adults

**DOI:** 10.3389/fneur.2024.1294689

**Published:** 2024-02-06

**Authors:** Paul Thisayakorn, Yanin Thipakorn, Saran Tantavisut, Sunee Sirivichayakul, Aristo Vojdani, Michael Maes

**Affiliations:** ^1^Department of Psychiatry, Faculty of Medicine, Chulalongkorn University, Bangkok, Thailand; ^2^Department of Orthopedics, Hip Fracture Research Unit, Faculty of Medicine, Chulalongkorn University, Bangkok, Thailand; ^3^Department of Medicine, Faculty of Medicine, Chulalongkorn University, Bangkok, Thailand; ^4^Immunosciences Lab Inc., Los Angeles, CA, United States; ^5^Cyrex Labs LLC, Phoenix, AZ, United States; ^6^Sichuan Provincial Center for Mental Health, Sichuan Provincial People’s Hospital, School of Medicine, University of Electronic Science and Technology of China, Chengdu, China; ^7^Key Laboratory of Psychosomatic Medicine, Chinese Academy of Medical Sciences, Chengdu, China; ^8^Department of Psychiatry, Medical University of Plovdiv, Plovdiv, Bulgaria; ^9^Research Institute, Medical University of Plovdiv, Plovdiv, Bulgaria; ^10^Kyung Hee University, Seoul, Republic of Korea; ^11^Cognitive Impairment and Dementia Research Unit, Faculty of Medicine, Chulalongkorn University, Bangkok, Thailand

**Keywords:** neuro-immune, leaky gut, bacterial translocation, inflammation, cytokines, biomarkers

## Abstract

**Introduction:**

Delirium is accompanied by immune response system activation, which may, in theory, cause a breakdown of the gut barrier and blood–brain barrier (BBB). Some results suggest that the BBB is compromised in delirium, but there is no data regarding the gut barrier. This study investigates whether delirium is associated with impaired BBB and gut barriers in elderly adults undergoing hip fracture surgery.

**Methods:**

We recruited 59 older adults and measured peak Delirium Rating Scale (DRS) scores 2–3 days after surgery, and assessed plasma IgG/IgA levels (using ELISA techniques) for zonulin, occludin, claudin-6, β-catenin, actin (indicating damage to the gut paracellular pathway), claudin-5 and S100B (reflecting BBB damage), bacterial cytolethal distending toxin (CDT), LPS-binding protein (LBP), lipopolysaccharides (LPS), *Porphyromonas gingivalis*, and *Helicobacter pylori*.

**Results:**

Results from univariate analyses showed that delirium is linked to increased IgA responses to all the self-epitopes and antigens listed above, except for LPS. Part of the variance (between 45–48.3%) in the peak DRS score measured 2–3 days post-surgery was explained by independent effects of IgA directed to LPS and LBP (or bacterial CDT), baseline DRS scores, and previous mild stroke. Increased IgA reactivity to the paracellular pathway and BBB proteins and bacterial antigens is significantly associated with the activation of M1 macrophage, T helper-1, and 17 cytokine profiles.

**Conclusion:**

Heightened bacterial translocation, disruption of the tight and adherens junctions of the gut and BBB barriers, elevated CDT and LPS load in the bloodstream, and aberrations in cell–cell interactions may be risk factors for delirium.

## Background

The estimated incidence of postoperative delirium in older adult patients with hip fractures is between 8.2%–24.0% (1–3). The major clinical characteristics of this acute and fluctuating neuropsychiatric syndrome are disturbances of attention, awareness, and other cognitive components (4). In hip fracture patients, delirium is associated with several adverse outcomes, such as an increased hospital stay, medical complications, poorer functional recovery, and an increased mortality rate (5–7). Older age, delirium history, premorbid dementia, multiple comorbidities, and functional dependency are common risk factors for postoperative delirium (2, 3, 8).

Hip fracture and surgery in older adults may initiate a complex neuro-pathophysiological process which leads to cognitive impairments and often delirium (9). The hypothesized pathophysiology of delirium comprises peripheral activation of the immune-inflammatory response system (IRS), neuroinflammation, oxidative stress, neurotransmitter dysregulation, and neural circuit disconnection (10, 11). Delirium in older adults following hip fracture surgery is associated with increased white blood cell numbers and neutrophil/lymphocyte ratio indicating aseptic inflammation (11). A recent meta-analysis shows that the delirium diagnosis is associated with a higher neutrophil/lymphocyte ratio in various critical care settings (12). Furthermore, we showed that delirium is characterized by IRS activation and a relative deficiency in the compensatory immunoregulatory system (CIRS), which prevents hyperinflammation (13). The increased IRS/CIRS ratio in delirium is indicated by increased macrophage M1; T helper (Th)1, and Th17 profiles, and a relative deficiency in Th2 and T regulatory (Treg) profiles (13).

There is now evidence that interactions between activated IRS pathways (e.g., M1 macrophage), breakdown of the BBB, and increased gut permeability play a role in neuropsychiatric disorders (14–17). Although previous studies have associated post-surgery delirium with BBB breakdown (18), there is no direct evidence that the breakdown of the paracellular pathway is associated with delirium. Nevertheless, there is some data on associations between gut microbiota diversities and postoperative delirium (19, 20). Few studies supported the hypothesis of an association between insomnia and abnormalities in the gut-brain axis (21, 22).

In the context of schizophrenia, notable correlations were identified between cognitive deficits or positive symptoms and biomarkers of IRS activation, including dysfunction of paracellular adherens junctions (e.g., IgA to E-cadherin and β-catenin) and tight junctions (e.g., IgA to occludin and zonulin), bacterial translocation (e.g., elevated IgA/IgM to Gram-negative bacteria), as well as disruption of the blood–brain barrier (e.g., increased IgA to occludin and β-catenin) (23, 24). A recent review and meta-analysis show that schizophrenia, major depression, bipolar disorder, and chronic fatigue syndrome are accompanied by indicants of leaky gut, with increased serum lipopolysaccharides (LPS) or antibodies directed to LPS of Gram-negative gut-commensal bacteria, LPS-binding protein (LBP), and zonulin (25, 26). In addition, increased IgG or IgA responses directed against *Helicobacter pylori* or *Porphyromonas gingivalis* may be associated with neurocognitive deficits in Alzheimer’s disease (27–29).

The hepatic secreted LBP may bind LPS in the systemic circulation, thereby forming an LPS-LBP complex, which consequently activates inflammatory cascades via the Toll-Like Receptor (TLR) 4 complex (30, 31). Some Gram-negative bacteria, including *Helicobacter* species, produce a bacterial cytolethal distending toxin (CDT), which is involved in IRS activation, host cell DNA intoxication, and apoptosis (32). Increased expression of LPS, the LPS-LBP complex, and CDT in the serum may indicate the breakdown of the gut barrier, with consequent increased translocation of bacterial antigens through the gut epithelium barrier into the lamina propria layer and the adjacent lymphatic and vascular systems (33–36). This bacterial translocation may occur via (a) the transcellular pathway and increased reactivity to actin may indicate this process (37, 38); and (b) the paracellular pathway, which comprises tight junctions (TJs) and adherens junctions (AJs) as major components (39, 40). Increased IgA/IgM/IgG responses to TJs (including zonulin, occludin, claudin-5/6) and AJs (including β-catenin) are biomarkers indicating the breakdown of the paracellular pathway (26).

Invasion of bacterial antigens through a dysfunctional intestinal barrier may trigger inflammatory cells inside the lamina propria layer to produce chemokines and pro-inflammatory cytokines, including tumor necrosis factor-α (TNF-α), interleukin (IL)-1, and IL-6, which may further expand the inflammatory signal to the systemic level (41). Conversely, systemic inflammation may damage the intestinal barrier and cause increased gut permeability due to, for example, increased levels of IL-1β, IL-6, and TNF-α (15, 26, 42). Reciprocal interactions between increased gut permeability and systemic IRS activation were demonstrated in disease models such as inflammatory bowel disease (43), cancer (44, 45), schizophrenia (46), depression (47, 48), and Alzheimer’s dementia (49, 50), but data in delirium is lacking.

Interestingly, the intestinal epithelial barrier and the BBB share some TJ and AJ-associated proteins such as occludin, claudin-5/6, β-catenin, and actin (24, 51). Some epitopes are more specific to the BBB, such as claudin-5 and S100 calcium-binding protein B (S100B) (52–54). Therefore, increased IgA/IgM/IgG responses to these epitopes may indicate increased damage to the gut (leaky gut) and BBB (leaky brain) barriers (24, 55). Despite recent evidence demonstrating increased IgA/IgM levels to gut and BBB breakdown epitopes in schizophrenia (24, 56, 57), depression (42, 58), autism (59), and Alzheimer’s disease (50, 60), no such data were reported in delirium.

Hence, the current study aims to examine (a) whether delirium severity in post-surgery patients with hip fracture is predicted by disturbances of the gut epithelial and the BBB as measured by using IgA/IgG responses to antigens and self-epitopes including LPS, LBP, CDT, zonulin, occludin, claudin-5, claudin-6, S100B, β-catenin, actin, *P. gingivalis* and *H. pylori;* and (b) whether these IgA/IgG responses are associated with the IRS response in post-surgery older adults.

## Methods

### Participants

A cohort of 59 elderly individuals with hip fractures who were admitted to the Hip Fracture Pathway Inpatient Care at King Chulalongkorn Memorial Hospital in Bangkok, Thailand, was enrolled in our study from June 2019 to February 2020. Patients who were 65 years of age or older and presented with a low-energy impact hip fracture, subsequently underwent surgery for the fracture, and were transferred to the surgery intensive care unit (SICU) or orthopedic wards, were included in the study. Individuals who met the following criteria were excluded from the study: major psychiatric illness (including schizophrenia, substance use disorders, bipolar disorder, psycho-organic disorders), a lifetime history of neuro-inflammatory and neurodegenerative diseases (including Alzheimer’s and Parkinson’s disease, multiple sclerosis), (auto) immune disorders (including psoriasis, systemic lupus erythematosus, inflammatory bowel disease, rheumatoid arthritis), coma, intracranial hemorrhage, pathologic fractures, and hip fractures from a high impact accidents. Individuals who have experienced remission of depression, modest neurocognitive disorders, or stroke without developing post-stroke disabilities within a year of the acute event may qualify for inclusion in the study.

### Clinical assessments

In addition to bedside interviews, sociodemographic and clinical information was gathered via electronic medical records. The cognitive status and delirium levels were evaluated at baseline, 24 h prior to the surgery. The cognitive status, delirium score, and diagnosis were evaluated daily for 3 days after the procedure. At the patient’s bedside, the Delirium Rating Scale, Revised-98-Thai Version (DRS-R-98-T) was utilized to assess the degree and manifestation of delirium until 3 days after the surgery (29, 30). This evaluation occurred on the evening of day zero, the day before the surgery, as well as in the morning and evening. The DRS-R-98-T demonstrates satisfactory inter-rater reliability and sensitivity and specificity in detecting delirium (29, 30). The severity of sleep–wake cycle disturbance was evaluated using the first item of the DRS-R-98-T (peak values of day 1 and 2), which ranged from normal (0 point) to severe (3 point) sleep disruption (61). As a result, scores two and three were merged into a single score (2), producing an ordinal variable with values of zero, one, and two. Data was gathered regarding the administration of benzodiazepines, opiates, anticholinergic medications, and psychiatric drugs, in addition to pertinent perioperative and postoperative clinical information including blood loss, operative duration, and the need for restraint because of agitation. Acute coronary events, arrhythmias, severe hypertension, and atrial fibrillation were among the cardiovascular complications obtained after surgery. The BMI was calculated by dividing the square of the subject’s height by their body weight (in kilograms).

The study protocol (registration number 528/61) was assessed and authorized by the institutional review board of the Faculty of Medicine, Chulalongkorn University. It adhered to the principles and procedures outlined in the International Guideline for the Protection of Human Subjects, the Belmont Report, the CIOMS Guideline, and the International Conference on Harmonization in Good Clinical Practice (ICH-GCP). Consent forms for the study were duly signed by every patient or their first-degree relatives.

### Determination of antibodies by ELISA

Aside from the clinical evaluation, venous blood samples were collected at 7 a.m. on day 0. Blood samples were stored at −80°C until thawed and then forwarded to the lab for IgA/IgG and cytokine/chemokine testing. IgA-and IgG antibodies to the epitopes were measured using ELISA methods. LPS from *Escherichia coli, Salmonella, Shigella, Klebsiella*, and *Pseudomonas*, LBP, and actin were purchased from Sigma-Aldrich (St. Louis, MO, United States), and occludin, zonulin, claudin-5, claudin-6, S100B, β-catenin, CDT, *P. gingivalis*, and S100B were synthesized by Bio-Synthesis® (Lewisville, TX, United States). *H. pylori* was purchased from the American Type Culture Collection® (Manassas, VA, United States). Jackson-Immuno Research® offered affinity pure goat anti-human IgA α-chain-specific and anti-human IgG, FC-specific (West Grove, PA United States). The IgG and IgA assays were performed as discussed previously (62). In brief: all antigens were prepared at a concentration of 1 mg/mL in 0.01 M phosphate buffer saline (PBS) pH 7.4. The optimal amount of each antigen was found to be one microgram in 100 microliters of 0.01 M carbonate buffer at pH 9.6, which was added to different wells of Costar ELISA plates. Plates were incubated at 25°C for 4 h, followed by overnight incubation at 4°C. In the next step, the unbound antigens were removed, and plates were washed 3 times with PBS containing 0.05% Tween 20, and 200 microliters of 2% bovine serum albumin (BSA) was added to block the non-coated regions of ELISA plate wells. Plates were kept at 4°C overnight, and after the BSA were washed, dried, and kept at 4°C until used. Calibrators, controls, and patients’ sera dilution at 1:50 for IgA and 1:100 for IgG in 0.01 M PBS pH 7.4 with 2% BSA and 0.05% Tween 20 were added to separate wells and incubated at room temperature for 1 h. Several wells contained all the reagents, but no serum was used to measure the background or blank ODs. After repeated washing and removing unattached serum proteins, alkaline phosphatase labeled anti-human IgA at 1:400 or anti-human IgG at 1:800 was added to separate sets of microwell plates and incubated for another hour. After repeating the washing procedure, adding 1 mg/mL of substrate para-nitrophenylphosphate, and incubating at room temperature for 30 min, a yellow color formed proportionately to the antibody concentration in the samples. The reaction was then halted with 60 microliters of 2 N NaOH, which created the endpoint color, which was measured at 405 nm with an ELISA reader. The antibody index was computed as follows: antibody index = (OD of sample − OD blank)/(OD of calibrator − OD of blank).

Based on the results we computed five IgA/IgG z-unit-based composite scores as (a) z transformation of IgA/IgG to LPS (z LPS) + z CDT + z *G. gingivalis* + z *H. pylori* (labeled IgA/IgG Bacterial, reflecting increased bacterial load); (b) z occludin + z zonulin + z claudin-6 (labeled IgA TJs, reflecting damage to the endothelial TJs); (c) z claudin-5 + z S100B (labeled IgA/IgG BBB, reflecting damage to the BBB especially when also IgA TJs are present), (d) z catenin + z actin (labeled IgA/IgG CATACT); and (e) z LPS + z LBP (labeled IgA/IgG LPS + LBP, reflecting increased LPS load in the plasma).

### Cytokine and chemokine assays

The methodology for analyzing cytokines and chemokines has been previously documented (13, 62). To summarize, the study employed the Bio-Plex ProTM Human Chemokine Assays manufactured by Bio-Rad Laboratories, Inc. in the United States of America. The Bio-Plex® 200 System (Carlsbad, California), was utilized to analyze the samples. 11.0% was the intra-assay CV for all analytes. In the data analysis, we utilized fluorescence intensities while subtracting the blank analyte values, as these intensities are a more reliable substitute for concentrations, particularly when examining numerous plates. The levels of IL-2, IL-10, IL-12, and IL-13 were omitted from the analyses focusing on a particular cytokine, because too many values were below the detection limit. Nonetheless, the values of those cytokines were included when creating immunological profiles because quantifiable levels of specific cytokines may contribute to the IRS/CIRS composites. The IRS and CIRS immune profiles, as well as the IRS/CIRS ratio (11, 62), were the primary immunological outcome determinants in this investigation. IRS was conceptualized as z-unit-based composite score based on z M1 (z IL-1β + z IL-6 + zTNF-α + z CXCL8 + z CCL3 + z IL-2 + z IL-12 + z interferon-γ + z IL-17). CIRS was conceptualized as z IL-4 + z IL-9 + z IL-13 + z IL-10 + z IL-1RA. The IRS/CIRS ratio was conceptualized as z IRS − z CIRS. Since IL-9 and IL-13 may have dual roles, we have recomputed the IRS/CIRS ratio without those two cytokines (labeled as IRS/CIRS2). There was a strong correlation between both IRS/CIRS and IRS/CIRS2 (r = 0.904, *p* < 0.001).

### Statistics

To ascertain relationships between sets of categorical data, the X^2^-test was utilized. Conversely, analysis of variance (ANOVA) was employed to investigate differences in scale variables between groups. The primary outcome analysis was the quantitative DRS-R-98 scale score, which was predicted by the explanatory variables, which are biomarkers measured 1 to 2 days prior. Pearson’s product moment correlation coefficients were computed to ascertain the relationships between the DRS scores and the IRS, CIRS, and IRS/CIRS data, as well as the IgA/IgG reactivity to self-epitopes and antigens. To examine the relationship between the dichotomized peak DRS scores on days 2–3 and the IgA/IgG responses on day 0 (which were entered as input variables), binary logistic regression and generalized estimating equation (GEE) analysis with repeated measurements were applied. We calculated the odds ratio (OR) with 95% confidence intervals and parameter estimates (B with SE values) for the logistic regressions; Nagelkerke values were utilized as pseudo-R^2^ effect sizes. While allowing for confounding variables, GEE was utilized to examine the relationships between the repeated DRS score measurements (days 2 and 3) and the IgA/IgG reactivities and DRS score on day 0. The study employed multiple regression analysis to investigate the relationship between predictors (such as IgA and IgG responses) and outcome variables (such as DRS scores), while controlling for confounding variables (such as age and sex). The effect size was determined using R^2^, and multivariate normality, collinearity, and multicollinearity were consistently assessed using Cook’s distance and leverage, tolerance and VIF, and the White and modified Breusch-Pagan tests for homoscedasticity, respectively. Furthermore, an automatic step-up method was implemented, incorporating values of 0.05 p-to-enter and 0.06 p-to-remove. All bootstrapped regression analyses were conducted using 5,000 samples; in cases where the results did not concur, the bootstrapped results are displayed. We accounted for the following variables in all regression analyses: gender, age, mild cognitive impairment, prior stroke, BMI, surgical duration, time to surgery, estimated blood loss during surgery, and use of deliriogenic medications. The statistical analysis was conducted using IBM SPSS for Windows version 28 (version 2022). A significance level of 0.05 was applied, and two-tailed tests were utilized. For numerous associations and comparisons, a False Discovery Rate (FDR) p correction was implemented. The *a priori* estimated sample size for a multiple regression analysis using G*Power analysis with an effect size of 0.30, an alpha of 0.05, a power of 0.80, and five predictors is around 49. Partial Least Squares (PLS) analysis using SmartPLS (SmartPLS) (23) was employed to examine whether the effects of age on the increases in the DRS score from baseline to 2–3 days later were mediated by IgA responses to different antigens.

## Results

### Sociodemographic and clinical data

The sociodemographic, clinical, and immune data of the study population are demonstrated in Table 1. To differentiate patients with increased DRS scores on days 2 and 3 from those with lower values, we computed the peak DRS values on day 2 and 3 and dichotomized the values using a visual binning method. Consequently, we examined two study groups, namely post-surgery patients with and without increased peak DRS values 2–3 days after surgery, using a cut-off value of ≥4. There were no significant differences between the two study groups in sex, education years, BMI, blood loss volume, duration from fall to hospital admission, hospitalization to surgery, or total duration of hospitalization. As expected (because higher age is a risk factor for delirium), patients in the high peak DRS group were significantly older than those in the low peak DRS group. The high-peak DRS group showed significantly higher IRS and IRS/CIRS scores than the low-peak DRS group, while there were no significant differences in CIRS scores between the groups.

**Table 1:** Socio-demographic, clinical and immune data in older adults divided into those with lower and higher peak Delirium Rating Scale, (DRS) scores on days 2 and 3 after surgery (peak DRS).

Variables	Low peak DRS (N = 36)	High peak DRS (*N* = 23)	F/χ2	df	*P*
DRS day0	2.28 (2.42)	3.48 (2.50)	3.36	1/57	0.072
DRS day1	1.86 (1.94)	4.87 (3.98)	15.06	1/57	<0.001
Peak DRS day2 + 3	2.15 (0.95)	6.78 (2.54)	54.69	1/57	<0.001
Age (years)	77.9 (7.7)	85.0 (5.9)	13.89	1/57	<0.001
Sex (F/M)	28/8	18/5	0.00	1	0.965
Education (years)	8.3 (5.4)	7.7 (6.4)	0.18	1/57	0.676
Body mass index (kg/m2)	22.02 (3.13)	21.51 (3.34)	0.33	1/57	0.567
Fall to hospital (hours)	2.2 (4.9)	1.3 (1.1)	0.61	1/57	0.439
Hospital to surgery (hours)	74.4 (76.2)	74.1 (58.5)	0.00	1/57	0.989
Length of stay (days)	9.2 (4.4)	12.1 (6.4)	4.29	1/57	0.043
Insomnia (0/1/2)	10/11/9	3/7/11	3.37	2	0.187
Blood loss (mL)	210.0 (115.6)	201.3 (107.0)	0.08	1/57	0.773
IRS (z scores)	−0.255 (0.938)	0.424 (1.071)	6.50	1/57	0.013
CIRS (z scores)	0.042 (0.941)	0.086 (1.192)	0.03	1/57	0.876
IRS/CIRS (z scores)	−0.258 (0.913)	0.392 (0.966)	6.69	1/57	0.012
IRS/CIRS2 (z scores)	−0.219 (0.921)	0.343 (1.145)	4.69	1/57	0.035

Results are shown as mean (SD) or as ratios. F, results of analyses of variance; χ2, results of analysis of contingency tables. IRS, z unit-based composite score reflecting the immune-inflammatory responses system; CIRS, z unit-based composite score reflecting the compensatory immune-regulatory system; Insomnia was scored as an ordinal variable, namely 0 (no symptoms), 1 (mild), and 2 (moderate to severe).

### Prediction of DRS score by biomarkers

Table 2 shows the results of binary logistic regression analyses with the high peak DRS group (reference group: low peak DRS group) as the dependent variable and the IgA bacterial, TJs, BBB, CATACT, and LPS + LBP composite scores. The high peak DRS group was significantly associated with baseline IgA scores across all five composite scores. Table 2 shows the results of GEE analyses with the DRS scores on days 2 and days 3 as dependent variables (repeated measures) and the IgA values to antigens on days 0 and 1 as predictors (repeated measures). As such, the IgA values at days 0 and 1 predicted the DRS values on days 2 and 3, respectively. We found that the 5 IgA values directed to the bacterial, TJs, BBB, CATACT, and LPS + LBP composite scores significantly predicted the DRS scores on days 2 and 3. The same table shows the results of the effects of the separate IgA values on the DRS values. We found that all IgA values directed to all epitopes, except LPS, were significantly associated with the DRS values some days later. Supplementary Figure 1 shows a clustered bar graph showing the IgA values for the 5 composites and all separate IgA values in post-surgery patients with low vs. high peak DRS scores. Supplementary Figure 2 shows that there were no significant differences in any of the IgG antibody values directed to the different epitopes.

**Table 2:** Results of binary logistic regression analysis (LR) and generalized estimating equations (GEE) with the Delirium Rating Scale (DRS), either as binary or continuous score, as dependent variables, and IgA responses to self-antigens as explanatory variables.

DRS Binary (LR)	B	SE	Wald (df = 1)	*p*	OR	95% CI
IgA Bacterial	0.286	0.105	7.45	0.006	1.33	1.08; 1.63
IgA Tight Junctions	0.266	0.098	7.42	0.006	1.31	1.08; 1.58
IgA BBB	0.531	0.186	8.15	0.004	1.70	1.18; 2.45
IgA β-catenin-actin	0.528	0.185	8.19	0.004	1.70	1.18; 2.44
IgA LPS + LPB	0.517	0.201	6.59	0.010	1.68	1.13; 2.49
**Peak DRS2 + 3 (GEE)**	**B**	**SE**	**Lower 95% CI**	**Higher 95% CI**	**Wald (df = 1)**	***p***
IgA Bacterial	1.271	0.3862	0.514	2.028	10.83	<0.001
IgA Tight junctions	1.303	0.3911	0.536	2.069	11.09	<0.001
IgA Blood brain barrier	1.297	0.3962	0.521	2.073	10.72	0.001
IgA β-catenin-actin	1.314	0.3954	0.539	2.089	11.05	<0.001
IgA LPS + LPB	1.197	0.3942	0.425	1.970	9.22	0.002
IgA LPS	0.819	0.4310	−0.026	1.664	3.61	0.057
IgA LPB	1.219	0.3445	0.544	1.894	12.52	<0.001
IgA Zonulin	1.027	0.4146	0.214	1.839	6.14	0.013
IgA Occludin	1.245	0.3883	0.484	2.006	10.28	0.001
IgA Claudin-5	1.325	0.3822	0.576	2.074	12.02	<0.001
IgA Claudin-6	1.315	0.3867	0.557	2.073	11.56	<0.001
IgA S100B	1.178	0.4006	0.393	1.963	8.65	0.003
IgA β-catenin	1.255	0.3846	0.501	2.009	10.65	0.001
IgA Actin	1.203	0.3855	0.447	1.958	9.73	0.002
IgA CDT	1.304	0.4025	0.515	2.093	10.50	0.001
IgA *P. gingivalis*	1.145	0.4133	0.335	1.955	7.67	0.006
IgA *H. pylori*	1.221	0.4055	0.426	2.015	9.06	0.003

LPS, lipopolysaccharide; LBP, lipopolysaccharide binding protein; CDT, cytolethal distending toxin; *P. gingivalis*, Porphyromonas gingivalis; *H. pylori*, *Helicobacter pylori*; z composite IgA scores, see assays for computation.

### Intercorrelation matrix between IgA responses, DRS, and IRS/CIRS data

Table 3 shows the intercorrelation matrix between the five IgA composite scores and the day 1, day 2, and peak DRS scores, as well as the IRS, CIRS, and IRS/CIRS values. The results indicate that there is no significant correlation between the five baseline IgA values and the DRS score on day 1, whereas there were highly significant associations with the peak DRS values. To examine the associations between the baseline IgA composite scores and the actual changes in the DRS scores from day 0 to days 2 and 3, we computed the residualized peak DRS values after regression on the DRS Day 0 values. These actual changes in DRS values were significantly associated with all five IgA composite scores. There were also significant correlations between the baseline IgA levels and the bacterial, TJs, BBB, CATACT, and LPS + LBP composite scores and the baseline IRS and IRS/CIRS scores, but less with the CIRS values. In addition, the IRS/CIRS2 ratio was significantly correlated with the bacterial (r = 0.414, *p* = 0.002), TJs (r = 0.438, *p* = 0.001), BBB (r = 0.356, *p* = 0.007), CATACT (r = 0.394, *p* = 0.003), and LPS + LBP (r = 0.390, *p* = 0.003) composite scores. All correlations remained significant after the FDR p correction.

**Table 3:** Intercorrelation matrix between the Delirium Rating Scale (DRS) scores, immune indices, and IgA responses to self-antigens.

Variables	Basal DRS	Peak DRS 2 + 3	Res peak DRS 2 + 3	IRS	CIRS	IRS/CIRS
IgA Bacterial	0.074 (0.590)	0.438 (<0.001)	0.461 (<0.001)	0.422 (0.001)	0.380 (0.021)	0.380 (0.004)
IgA Tight junctions	0.089 (0.516)	0.436 (<0.001)	0.491 (<0.001)	0.473 (<0.001)	0.339 (0.011)	0.414 (0.002)
IgA Blood brain barrier	0.077 (0.573)	0.457 (<0.001)	0.490 (<0.001)	0.462 (<0.001)	0.343 (0.010)	0.392 (0.003)
IgA β-catenin-actin	0.158 (0.246)	0.425 (0.001)	0.483 (<0.001)	0.407 (0.002)	0.193 (0.155)	0.344 (0.009)
IgA LPS + LPB	0.089 (0.514)	0.378 (0.004)	0.399 (0.002)	0.380 (0.004)	0.232 (086)	0.385 (0.003)

Basal DRS, DRS score on day 0; peak DRS2 + 3, peak DRS scores on days 2 and 3 after surgery; res peak DRS, residualized changes in peak DRS2 + 3 scores after partialling out the effects of basal DRS (dubbed the residualized peak DRS2 + 3); IRS, z unit-based composite score reflecting the immune-inflammatory responses system; CIRS, z unit-based composite score reflecting the compensatory immunoregulatory system; z composite IgA scores, see assays for computation.

While all IgA responses were correlated with the peak DRS and residualized peak DRS scores, not one of the five IgG composites was correlated with the clinical scores, even without FDR p correction. On the other hand, the IRS/CIRS ratio was significantly correlated with IgG CATACT (r = 0.299, *p* = 0.025), LBP + LPS (r = 0.361, *p* = 0.006), TJs (r = 0.342, *p* = 0.010) and BBB (t = 3.11, *p* = 0.020) composite scores. These correlations remained significant after the FDR p correction. Insomnia was not significantly correlated with IgA or IgG levels to bacterial, TJ, BBB, CATACT, and LPS + LBP composites, even without FDR p correction.

### Results of multiple regression analysis

**Figure 1:**
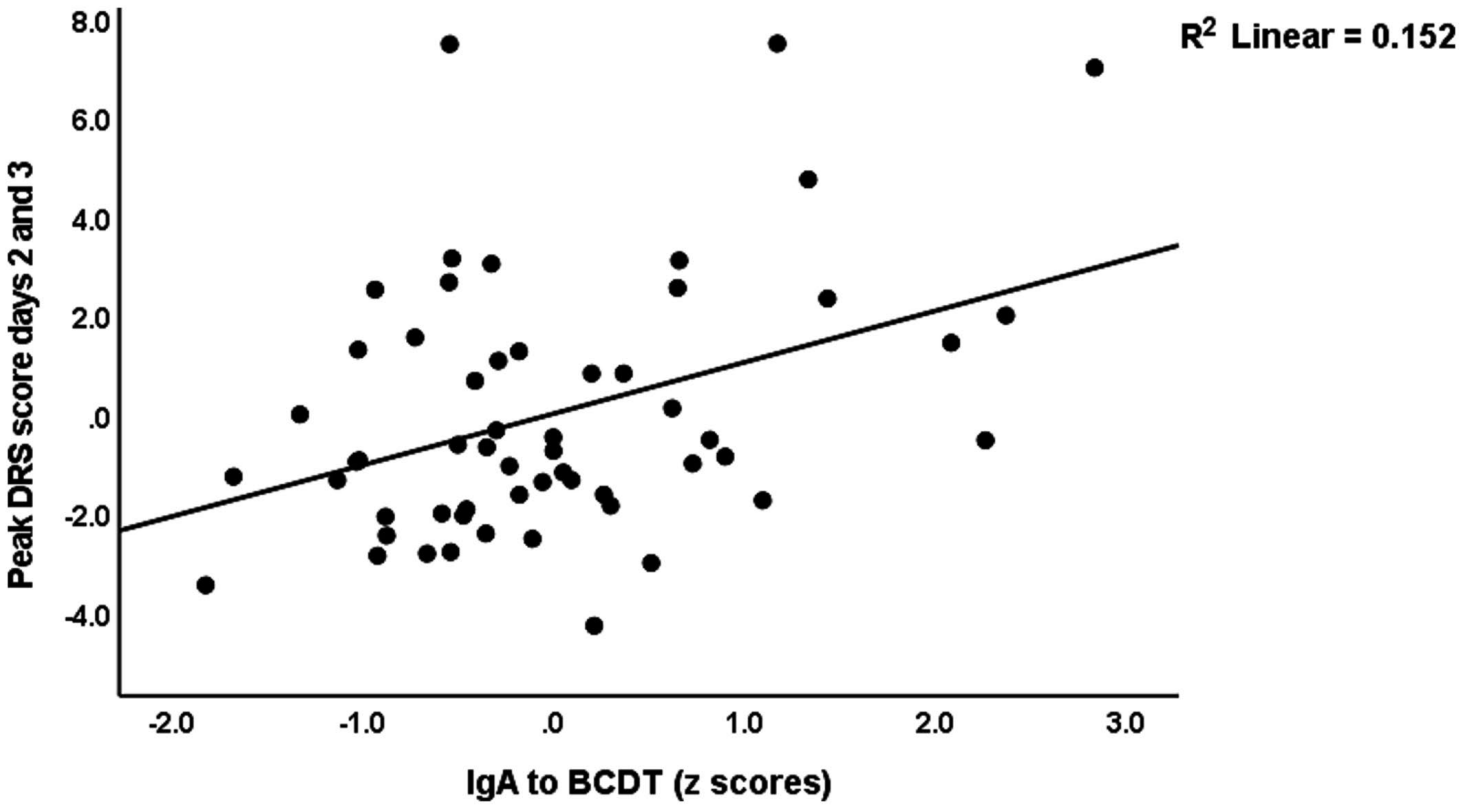
Partial regression of peak Delirium Rating Scale (DRS) scores on IgA to bacterial cytolethal distending toxin (BCDT).

**Figure 2:**
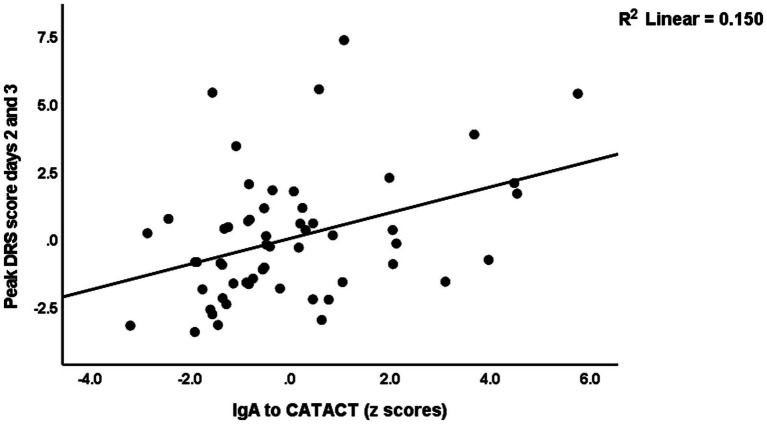
Partial regression of peak Delirium Rating Scale (DRS) scores on IgA to the β-catenin-actin (CATACT) complex.

Table 4 shows the results of multiple regression analyses with the DRS scores as dependent variables and the IgA composites as well as the DRS values on days 0 and 1, and demographic data and known risk factors for delirium (age, sex, previous stroke) as explanatory variables. Regression #1 shows that 48.3% of the variance in the peak DRS scores could be explained by the regression on IgA to CDT, DRS days 0, and previous stroke (all three positively associated). Figure 1 shows the partial regression of peak DRS values on IgA to CDT. Regression #2 examines the same variables, except that DRS days 0 and 1 were excluded from the analysis. This regression shows that 47.1% of the variance in the peak DRS score was explained by the regression on IgA claudin-6, a history of stroke, age, and SSRI treatment before admission; 30.1% of the variance in the DRS score on day 0 could be explained by age and SSRI. Regression #4 found that 45.0% of the variance in the peak DRS scores is associated with DRS Day 1, IgA CATACT, and a history of previous stroke (all three are positively associated). Figure 2 shows the partial regression of peak DRS scores on IgA to the CATACT composite. Deleting IgA to CDT and CATACT showed that the IgA BBB composite was the third most significant explanatory variable that, together with age and previous stroke, explained 39.6% of the variance in the peak DRS values.

**Table 4:** Results of multiple regression analyses with the peak Delirium Rating Scale, Revised-98-Thai version (DRS) scores on days 2 and 3 post-surgery, DRS score on day 1, and immune indices as dependent variables.

Dependent variables	Explanatory variables	B	t	p	F model	df	p	R^2^
# 1 Peak DRS2 + 3	Model				16.51	3/53	<0.001	0.483
	DRS Day 0	0.415	4.11	<0.001				
	IgA CDT	0.429	4.29	<0.001				
	Previous stroke	0.280	2.77	0.008				
#2 Peak DRS2 + 3	Model				11.57	4/52	<0.001	0.471
	IgA Claudin-6	0.450	4.27	<0.001				
	Stroke	0.281	2.73	0.009				
	Age	0.331	3.18	0.003				
	SSRI	0.243	2.30	0.025				
#3 DRS Day 0	Model				11.62	2/54	<0.001	0.301
	Age	0.484	4.22	<0.001				
	SSRI	0.329	2.87	0.006				
#4 Peak DRS2 + 3	Model				14.44	3/53	<0.001	0.450
	DRS Day-1	0.448	4.31	<0.001				
	IgA CATACT	0.345	3.36	0.001				
	Previous stroke	0.231	2.25	0.29				
#5 Peak DRS2 + 3	Model			11.57		3/53	<0.001	0.396
	IgA BBB	0.382	3.47	<0.001				
	Previous stroke	0.324	3.03	0.004				
	Age	0.311	2.82	0.007				
#6 IRS/CIRS	Model				6.99	3/53	<0.001	0.284
	IgA Claudin-6	0.342	2.86	0.006				
	Previous stroke	0.276	2.37	0.021				
	Age	0.247	2.07	0.044				
#7 IRS/CIRS	Model				6.96	3/53	<0.001	0.283
	IgA TJs	0.341	2.84	0.006				
	Previous stroke	0.272	2.33	0.023				
	Age	0.244	2.03	00.47				
#8 IRS/CIRS	Model				9.68	4/51	<0.001	0.432
	IgA TJs	0.296	2.66	0.011				
	IgG LPB + LPS	0.375	3.40	0.001				
	Age	0.333	2.61	0.012				
	Previous stroke	0.244	2.30	0.026				

CDT, cytolethal distending toxin; LBP, lipopolysaccharide binding protein; LPS, lipopolysaccharide; BBB, blood brain barrier; TJs, tight junctions; IRS, z unit-based composite score reflecting the immune-inflammatory responses system; CIRS, z unit-based composite score reflecting the compensatory immunoregulatory system.

**Figure 3:**
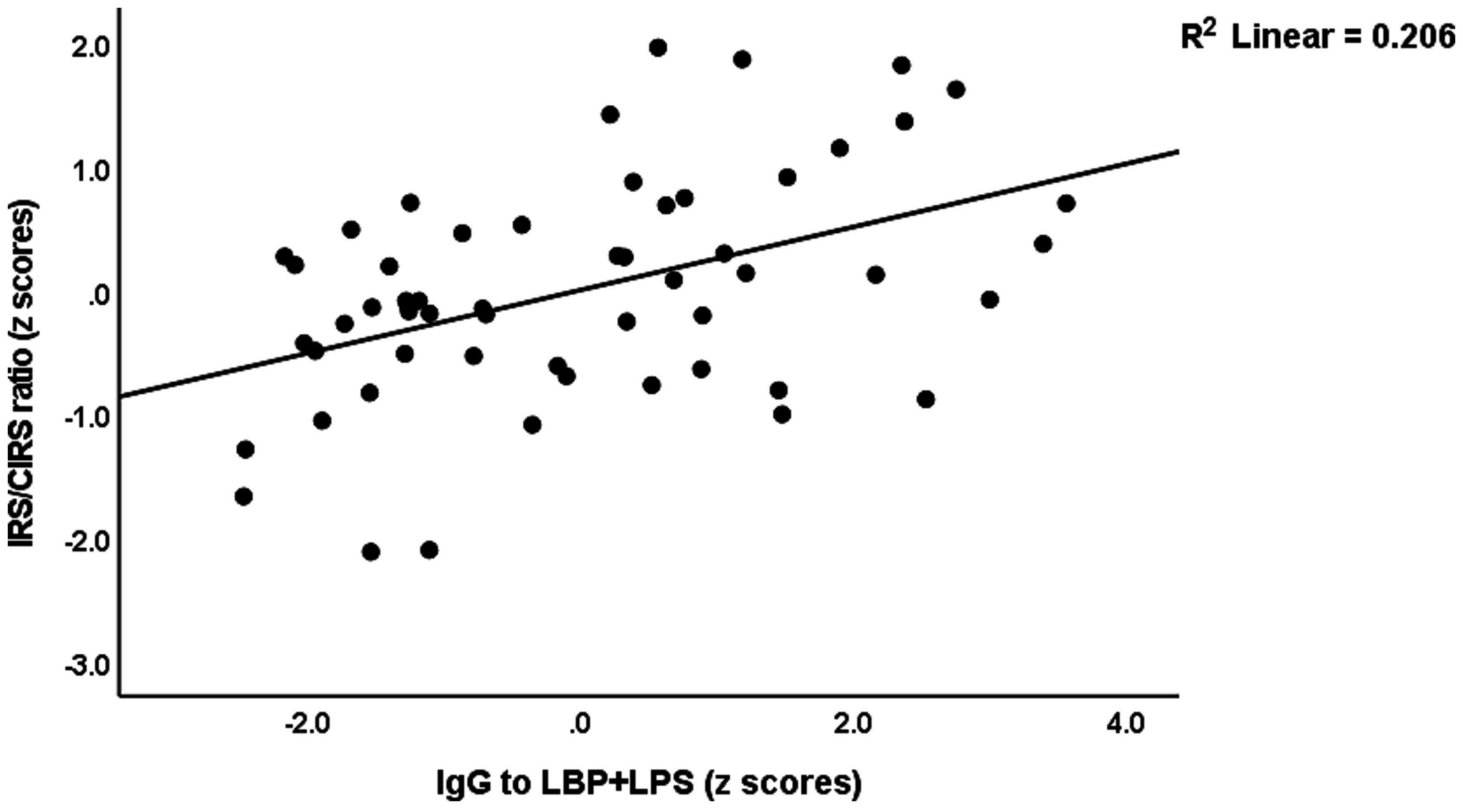
Partial regression of the immune response system (IRS)/compensatory immunoregulatory system (CIRS) on IgA to the lipopolysaccharide (LPS)—LPS-binding protein (LBP + LPS) complex.

The same table shows the results of multiple regression analyses with the IRS/CIRS values as the dependent variables and IgA/IgG responses to different epitopes as the explanatory variables. Entering the IgA responses to the separate epitopes (regression #6) showed that 28.4% of the variance in the IRS/CIRS ratio is explained by IgA claudin-6, previous stroke, and age (all positively associated). The regression of IRC/CIRS2 on the same variables showed that claudin-6 was a significant predictor (β = 0.404, t = 3.21, *p* = 0.002). Entering the five IgA composite scores (regression #7) shows that 28.3% of the variance in the IRS/CIRS ratio is explained by IgA to TJs, previous strokes, and age as explanatory factors. Entering IRS/CIRS2 as a dependent variable showed that TJ was the most significant composite (β = 0.431, t = 3.51, *p* = 0.001), whereas age and stroke were not significant. Finally, in regression #7, we have also added the IgG responses and found that 43.2% of the variance in the IRS/CIRS ratio (regression #8) was explained by IgG to LBP + LPS day 0 and the same variables as in regression #7. Figure 3 shows the partial regression of the IRS/CIRS ratio on IgG to LBP + LPS. Entering IgG to CATACT (β =0.266, t = 2.33, *p* = 0.024), and TJs (β =0.270, t = 2.31, *p* = 0.025) instead of LBP + LPS in regression #8 showed that these IgG responses were also significant.

**Figure 4:**
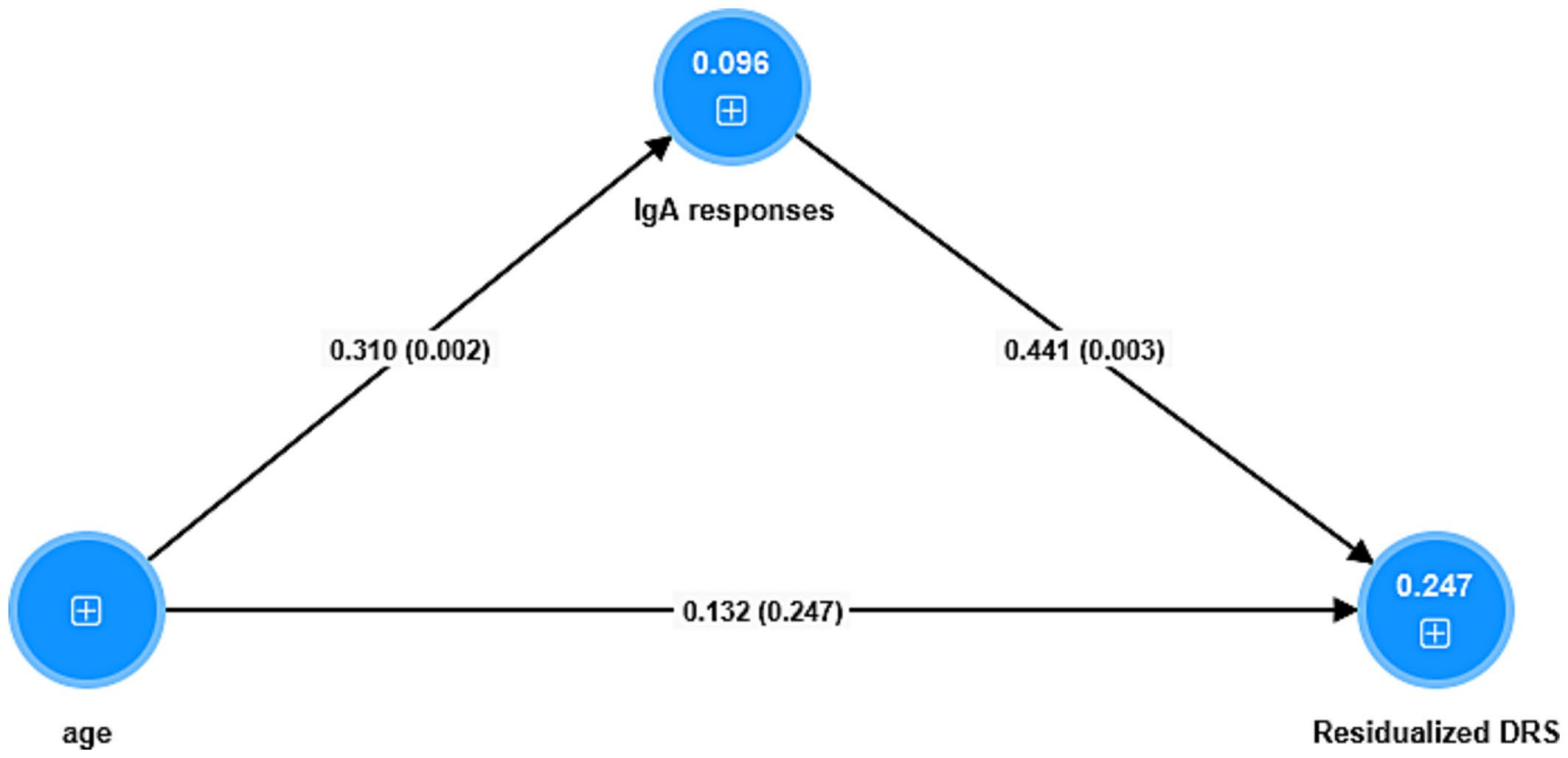
Results of partial least squares analysis. The outcome is the residualized (res) endpoint Delirium Rating Scale (DRS) score value (baseline values covaried out). The predictors are a factor extracted from 5 IgA responses (labeled IgA responses) to antigens, and age whereby the latter is allowed to predict the IgA responses. Shown are the pathway coefficients (with *p*-values). The figures in the blue circles denote the explained variances. The IgA responses combine blood brain barrier, lipopolysaccharides, lipoprotein binding protein, bacterial load in the bloodstream, catenin and actin, and tight junctions proteins.

Figure 4 shows the results of a mediation PLS analysis with the changes in the DRS score from baseline to a few days later as the dependent variable and the IgA responses (entered as a factor extracted from the 5 IgA indices; labeled IgA responses) as the mediator between age and the changes in the DRS score. The IgA responses factor shows adequate validity (Cronbach’s alpha = 0.92; explained variance = 0.81, all loadings > 0.66) and the model quality fit data are adequate (SRMR = 0.036). There was a significant specific indirect effect of age on the DRS score that was completely mediated by the IgA responses (t = 1.98, *p* = 0.048). There was a significant direct effect of age on the IgA responses, and a significant total effect of age on the DRS score (t = 2.65, *p* = 0.008).

## Discussion

### IgA biomarkers of delirium severity

The first major finding of this study is that delirium and DRS scores are predicted by IgA responses to bacterial, LPS-LBP, TJ, BBB breakdown, and CATACT in older adults with hip fractures. To our knowledge, this is the first study demonstrating an association between delirium severity and IgA responses to LPS, LPB, CDT, *H. pylori*, zonulin, occludin, claudin-5, claudin-6, β-catenin, and actin. These findings extend those of previous publications showing an association between delirium and *P. gingivalis* (63), and S100B (64, 65).

Older adults who were exposed to hip fracture surgery, trauma with connective tissue injury, perioperative state, ICU stay, dehydration and hypoxemia, infection, and metabolic impairment are more vulnerable to developing delirium syndrome, especially when they have neurocognitive impairments (6). These precipitating factors may aggravate various upstream processes of circadian dysregulation (66), HPA axis dysregulation (67), oxidative stress (68), and neuro-inflammation (69), which consequently and interactively may cause neurotransmitter dysregulation (70) and neuronal network disconnection (71), which are clinically expressed as delirium (9, 10, 72). Therefore, the immune findings from this study indicate that, apart from the above risk factors, bacterial translocation (LPS-LPB) (73, 74), gut-blood–brain barrier dysfunctions [zonulin (75), occludin and claudin (76, 77)] are involved, as indicated by animal models and human studies.

Interestingly, the current immune findings in delirium partly overlap with previous findings in schizophrenia. Thus, in schizophrenia, several studies reported significant associations with increased bacterial translocation (78–80), breakdown of transcellular and paracellular tight/adherens junction barriers (23, 81, 82), as well as the BBB (24, 83). Moreover, our study demonstrated a significant association between increased levels of IgA in *P. gingivalis*, *H. pylori*, and CDT (and their z composite score) and delirium severity. *P. gingivalis* is a Gram-negative bacterium found in the oral mucosa and is reported to be involved with periodontitis and low-grade systemic inflammation-related diseases such as atherosclerosis, diabetes, cancer, depression, and schizophrenia (84, 85). Similarly, *H. pylori* infection directly contributes to localized inflammation and is indirectly associated with cardiovascular disease, metabolic syndrome, autoimmune diseases, and Parkinson’s disease (86, 87). Both bacteria were evidently associated with the development of Alzheimer’s disease and other dementia syndromes (88–90).

The results of this study also demonstrated a significant association between delirium and IgA responses to the LPS + LPB complex and CDT. LPS (endotoxin) and its systemic response protein secreted from the liver (LBP), jointly form the LPS-LPB complex (91). Therefore, these IgA responses are indicants of increased bacterial load in peripheral blood (25). Moreover, the LPS-LBP complex in peripheral blood may further activate the innate immune system and pro-inflammatory cytokines via activating the TLR-4 pathway (34, 35). Elevated CDT may dysregulate the immune-inflammatory system by breaking the intra-cellular and intra-nucleus cascades, leading to an apoptotic process in epithelial barrier cells and acquired immune lymphocytes, and activating pro-inflammatory cytokine-secreting macrophages (92, 93).

Our study also shows significant correlations between IgA directed to claudin-5, occludin, zonulin, and tight junctions, and increased DRS-R-T scores. Tight junctions are located at the apical intercellular area of the intestinal epithelial cell lines, and function as a barrier between the intraluminal and systemic parts of the gastrointestinal tract (94). Claudins are transmembrane proteins located at the inner and outer rims of the tight junctions and coupled with occludins, which maintain the structural integrity of the tight junctions, and function as gatekeepers of the paracellular route (95, 96). Zonulin, on the other hand, induces the breakdown of the tight junctions, and increased zonulin levels indicate a leaky gut (97).

Adherens junctions (AJs) are located underneath the tight junction in the paracellular pathway and comprise the transmembrane E-cadherin protein, cytoplasmic alpha-and beta-catenin binding proteins, and cytoskeletal actin components (98). AJs support inter-epithelial barrier functions, maintain cell and tissue architecture, cytoskeletal regulation, and cell signaling and gene transcription (99, 100). All in all, these IgA findings imply a leaky gut, namely the breakdown of TJs and AJs and increased translocation of common Gram-negative or Gram-positive microbiota or their detrimental antigens into the systemic circulation. These mechanisms are frequently associated with TLR-4 complex activation and increased inflammatory signaling, which may result in systemic diseases (31, 101).

It should be stressed that the delirious patients in our study showed significantly increased IgA reactivity to S100B, and claudin-5, which are specific products of BBB breakdown. As such, these findings, together with increased IgA reactivity to TJ and AJ antigens, not only indicate gut barrier impairments but also represent damage to the BBB. Significant evidence of the disruption of the gastrointestinal (gut) and blood–brain barriers (BBB) has been observed in individuals with neurocognitive impairments, autism, and schizophrenia (24, 42, 59, 102). Notably, certain conditions that increase the likelihood of delirium are also linked to both increased intestinal permeability and the translocation of LPS or bacteria from the gut into the bloodstream causing tissue damage (103), bone fracture (104, 105), aging (106), stroke (107), sepsis (108, 109), liver failure (110, 111), uremia (112, 113), alcohol (114, 115), malnutrition (116, 117), and psychological stressors (118, 119). Furthermore, these risk variables were also linked to BBB disintegration (120–127), which is considered a prevalent and significant contributing factor to delirium (128).

### IgA and IgG reactivity, IRS/CIRS, and cell–cell interactions in delirium

The second major finding of this study is that IRS activation in the postoperative period is strongly associated with IgA responses to paracellular and BBB composites and bacterial antigens, and in addition to IgG levels directed to TJs, and LBP + LPS and β-catenin/actin. We previously reported that the onset and severity of delirium are significantly correlated with IRS activation, including increased M1 (with IL-6, IL-8, and TNF-α), Th1, Th17, and T cell growth profiles (13). Consequently, our results indicate that leaky barriers and bacterial antigens increase the risk of delirium in part by activating the IRS. It is interesting to note that IgA/IgG responses observed in our study were strongly associated with IRS activation and did not impact the CIRS.

Moreover, natural polyreactive IgA antibodies (PABs) such as those determined here may induce immune-inflammatory responses and contribute to inflammatory disorders and autoimmunity (28, 129). Furthermore, PAB administration to the brain may cause damage to neuronal circuits (65), and low-affinity PABs may serve as precursors for high-affinity pathogenic Abs (66). Therefore, our data imply that increased IgA reactivity may further enhance IRS activation (130–133). Moreover, increased IgA directed to β-catenin may also implicate cadherin signaling, cell–cell interactions, and thus intracellular signal transduction, cell proliferation, cell migration, and apoptosis (134).

## Limitations

A limitation of this study could be the smaller sample size of older adults with hip fractures. Nevertheless, the *a priori* estimated sample size was at least 49 when considering an effect size of 0.30, an alpha of 0.05, and a power of 0.80. Furthermore, the actual power for the regression analyses performed in this study (see Table 4) with the DRS scores as dependent variables, alpha = 0.05, *n* = 59, and 3 predictors (see Table 1) was 0.999, indicating a well-powered study. Future studies should be carried out to replicate these findings in other countries and cultures. An open question is whether these biomarkers are traits (risk factors of post-injury delirium) or state biomarkers of delirium. Therefore, future research should measure these biomarkers before trauma and surgery and again after surgery. It would also be interesting to assess the enterotype of gut dysbiosis in delirium to examine whether gut dysbiosis contributes to delirium via the increased leaky gut.

Another concern could be that BBB breakdown composite markers measured in the blood may be insufficient to conclude that increased BBB permeability is a risk factor for delirium. Nevertheless, many different findings in patients with delirium indicate that BBB breakdown is involved: (a) damage to the most important proteins of the paracellular pathway (as indicated by our findings) plays a role in BBB disruption (135). Furthermore, alterations in the tight junction protein complexes (as detected in our study) are known to result in increased paracellular permeability leading to increased BBB permeability (136). (b) peripheral inflammation with increased levels of some pro-inflammatory cytokines as detected in delirium (13) is known to lead to BBB disruption (137, 138). (c) Previously, we have shown increased autoimmunity against neuronal self-epitopes indicating glial fibrillary acidic protein, neurofilament protein, glial fibrillary acidic protein, myelin basic protein, myelin oligodendrocyte glycoprotein, metabotropic glutamate receptors mGluRs 1 and 5, N-Methyl-D-Aspartate receptor (NMDAR) GLU_1_ (NR_1_) and GLU_2_ (NR_2_) (62). In addition, these indicants of damage to neural tissue epitopes are associated with signs of peripheral immune activation and severity of delirium (62). Future research should measure other specific biomarkers of BBB breakdown in delirious patients, such as increased leakage of gadolinium as assessed using magnetic resonance imaging, increased CSF plasminogen and fibrinogen, and increased peripheral blood neuron-specific enolase (NSE) (139, 140).

Lastly, the difference in age between patients with high and low peak DRS scores and the knowledge that IgA levels may increase with age (141) may be perceived by some as a limitation of this study. Nevertheless, our study is not a case–control study, but a cohort study that examines the predictive effects of baseline risk factors (IgA levels and age, and other) on the onset of delirium 2–3 days later. As such, the primary outcome of this cohort study is the regression of the changes in the DRS scores on the basal risk factors. Since age is a risk factor of delirium (13), it is natural that in our cohort study, those with higher DRS scores have a higher age. To identify the risk factors for increased DRS scores, including IgA responses to self-epitopes, and bacterial antigens, age, and comorbidities, we conducted a prospective cohort study with patients exposed to the same injury. Therefore, the primary analyses of this study are the multiple regression analyses that characterize the risk factors for delirium severity and not the comparison between patients with low and high DRS scores. These are only displayed to illustrate the mean values of the measured variables. It should be noted that matching the delirium and control groups by age would result in selection bias due to the partial restriction imposed by group selection. This methodology would result in gains or losses in multiple regression analyses and, by implication, imprecision. Any such selection or matching based on age has the potential to introduce substantial bias into the regression results (13). By employing mediation analysis, we investigated the relationships between age, IgA responses, and changes in the DRS score. Our findings indicate that IgA responses play a substantial mediating role in the effects of age on changes in the DRS score. In other words, our prospective cohort study found that higher IgA responses can be considered as biomarkers of post-surgery delirium in old adults. It is important to replicate and validate our cohort study in different nations and cultures. Additionally, case–control studies should investigate whether these potential predictive biomarkers may be used as diagnostic biomarkers.

## Conclusion

Aberrations in the tight and adherens junctions of the paracellular pathway of the gut and BBB barriers, increased bacterial translocation and LPS and DCT load in the systemic circulation, and cell–cell interactions are identified as risk factors of delirium in older adults after hip fracture surgery. IgA/IgG reactivity to the antigens measured here may contribute to IRS activation, which is another pathophysiological factor leading to delirium and which could mediate at least in part the effects of IgA/IgG responses on delirium. Consequently, leaky gut, translocation of bacterial antigens, IRS activation, and BBB disruption are new drug targets to treat and potentially prevent delirium. The data from animal and human studies demonstrate the use of antioxidants, including zinc and glutamine, norfloxacin, infliximab, tofacitinib, CKD-506, and larazotide acetate to restore the leaky gut barrier and prevent or attenuate bacterial translocation (142–146). Minocycline, raparixin, atorvastatin, melatonin, and mesenchymal stromal cell therapy may promote BBB restoration in various neurological conditions (147, 148).

## Data availability statement

The raw data supporting the conclusions of this article will be made available by the authors, without undue reservation.

## Ethics statement

The studies involving humans were approved by the Institutional Review Board of the Faculty of Medicine, Chulalongkorn University, Bangkok, Thailand (registration number 528/61). The studies were conducted in accordance with the local legislation and institutional requirements. The participants provided their written informed consent to participate in this study.

## Author contributions

PT: Conceptualization, Data curation, Funding acquisition, Investigation, Methodology, Project administration, Resources, Validation, Visualization, Writing – original draft, Writing – review & editing. YT: Data curation, Investigation, Methodology, Project administration, Resources, Validation, Writing – review & editing. ST: Conceptualization, Investigation, Methodology, Project administration, Resources, Supervision, Writing – review & editing. SS: Data curation, Formal Analysis, Investigation, Methodology, Resources, Software, Validation, Writing – review & editing. AV: Data curation, Investigation, Methodology, Resources, Software, Writing – review & editing. MM: Conceptualization, Data curation, Formal Analysis, Methodology, Resources, Software, Supervision, Visualization, Writing – original draft, Writing – review & editing.
