# Supplementary material for: Increased IgA-mediated responses to the gut paracellular pathway and blood–brain barrier proteins predict delirium due to hip fracture in older adults
Source: Front Neurol. 2024 Feb 6;15:1294689. doi: 10.3389/fneur.2024.1294689 (PMC10876854; doi:10.3389/fneur.2024.1294689)
Supplement: Supplementary file 1 [file Image_1.pdf]

ELECTRONIC SUPPLEMENTARY FILE (ESF)

Breakdown of the gut paracellular and blood-brain barrier and disorders in the β-catenin- actin complex are risk factors of delirium due to hip fracture in older adults.

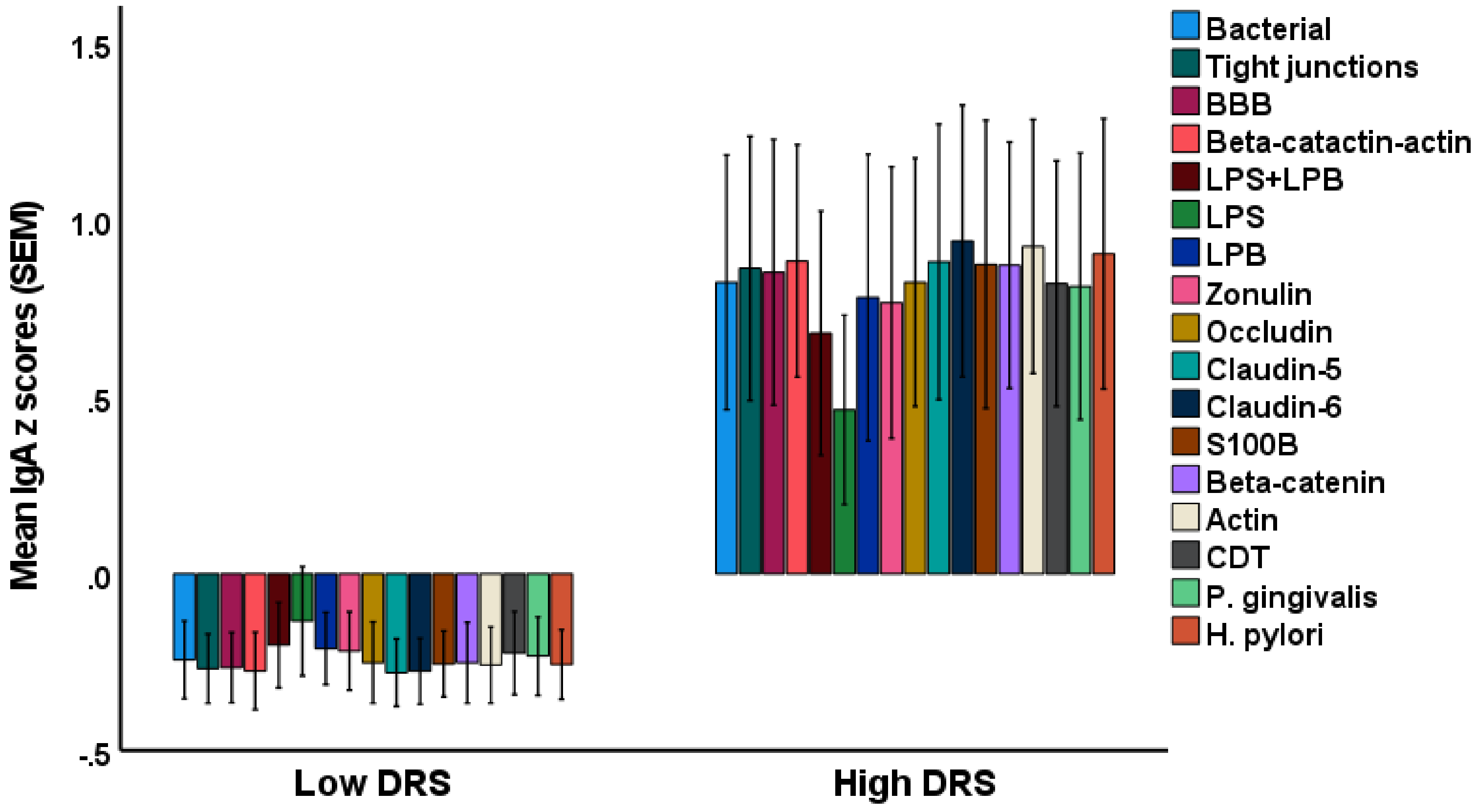

Supplementary Figure 1. IgA responses to self-antigens in patients with high versus low responses in the Delirium Rating Scale (DRS) scores, 2-3 days after surgery. LPS: lipopolysaccharide; LBP: lipopolysaccharide binding protein; CDT: cytolethal distending toxin; *P. gingivalis*: *Porphyromonas gingivalis*; *H. pylori*: *Helicobacter pylori*; z composite IgA scores: see assays for computation. All significant at $p<0.05$ (except LPS)

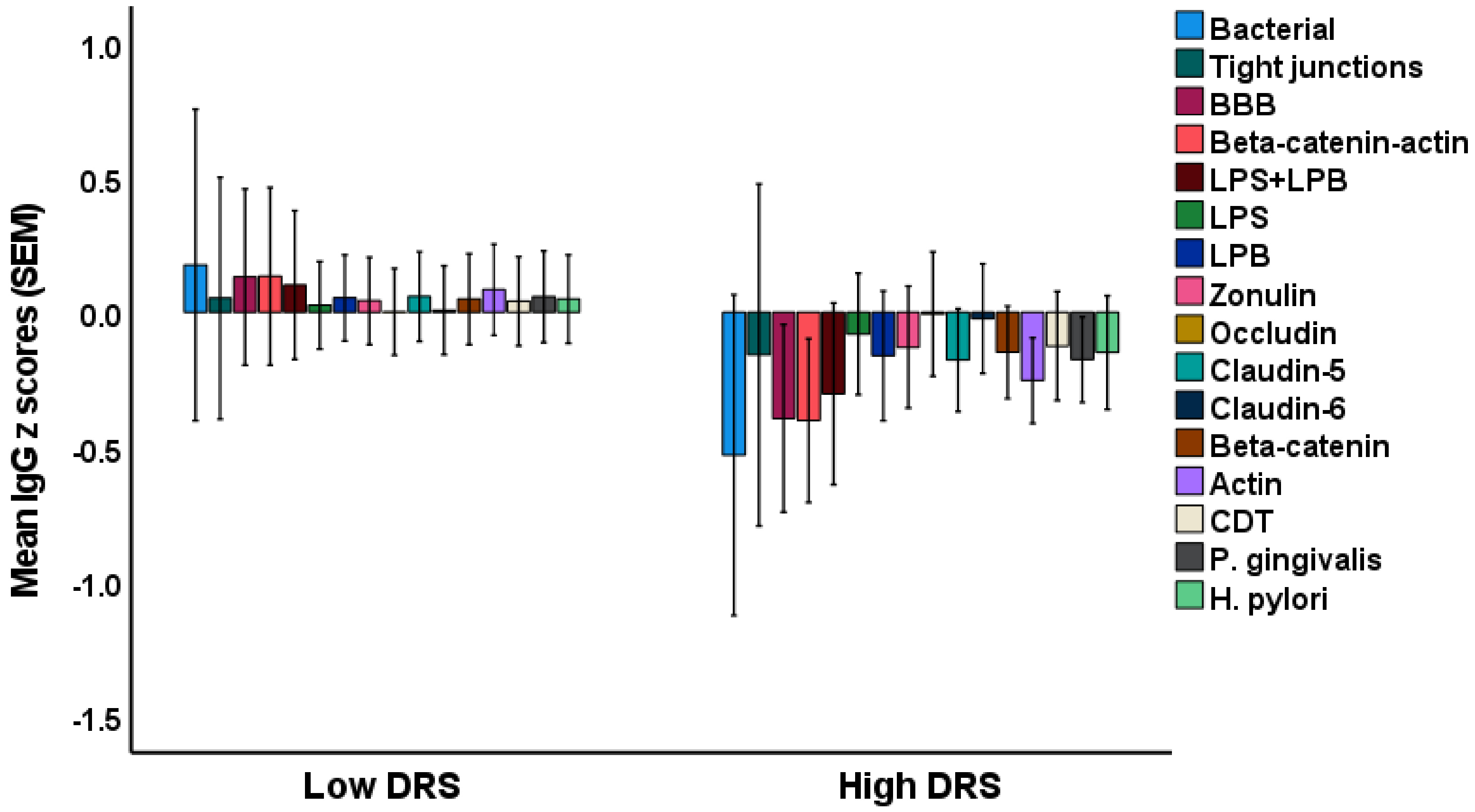

Supplementary Figure 2. IgG responses to self-antigens in patients with high versus low responses in the Delirium Rating Scale (DRS) scores, 2-3 days after surgery. LPS: lipopolysaccharide; LBP: lipopolysaccharide binding protein; CDT: cytolethal distending toxin; *P. gingivalis: Porphyromonas gingivalis*; *H. pylori: Helicobacter pylori*; z composite IgA scores: see assays for computation. All not significant ($p<0.05$)
